# uPA is upregulated by high dose celecoxib in women at increased risk of developing breast cancer

**DOI:** 10.1186/1471-2407-8-298

**Published:** 2008-10-15

**Authors:** Wenyi Qin, Weizhu Zhu, John E Hewett, George Rottinghaus, Yin-Chieh Chen, John T Flynn, Beth Kliethermes, Ferdinando Mannello, Edward R Sauter

**Affiliations:** 1 Department of Surgery, University of North Dakota, Grand Forks, ND, USA; 2 Departments of Biostatistics, University of Missouri, Columbia, MO, USA; 3 Veterinary Medical Diagnostics, University of Missouri, Columbia, MO, USA; 4 Institute of Histology and Laboratory Analysis, Thomas Jefferson University, Philadelphia, PA, USA; 5 Department of Physiology, Thomas Jefferson University, Philadelphia, PA, USA; 6 University "Carlo Bo", Urbino 61029, Italy

## Abstract

**Background:**

While increased urokinase-type plasminogen activator (uPA) expression in breast cancer tissue is directly associated with poor prognosis, recent evidence suggests that uPA overexpression may suppress tumor growth and prolong survival. Celecoxib has been shown to have antiangiogenic and antiproliferative properties. We sought to determine if uPA, PA inhibitor (PAI)-1 and prostaglandin (PG)E_2 _expression in nipple aspirate fluid (NAF) and uPA and PGE_2 _expression in plasma were altered by celecoxib dose and concentration in women at increased breast cancer risk.

**Methods:**

NAF and plasma samples were collected in women at increased breast cancer risk before and 2 weeks after taking celecoxib 200 or 400 mg twice daily (bid). uPA, PAI-1 and PGE_2 _were measured before and after intervention.

**Results:**

Celecoxib concentrations trended higher in women taking 400 mg (median 1025.0 ng/mL) compared to 200 mg bid (median 227.3 ng/mL), and in post- (534.6 ng/mL) compared to premenopausal (227.3 ng/mL) women. In postmenopausal women treated with the higher (400 mg bid) celecoxib dose, uPA concentrations increased, while PAI-1 and PGE_2 _decreased. In women taking the higher dose, both PAI-1 (r = -.97, p = .0048) and PGE_2 _(r = -.69, p = .019) in NAF and uPA in plasma (r = .45, p = .023) were correlated with celecoxib concentrations.

**Conclusion:**

Celecoxib concentrations after treatment correlate inversely with the change in PAI-1 and PGE_2 _in the breast and directly with the change in uPA in the circulation. uPA upregulation, in concert with PAI-1 and PGE_2 _downregulation, may have a cancer preventive effect.

## Background

Cancer cell invasion and metastasis requires the degradation of the extracellular matrix (ECM) and basement membrane. This process is accomplished by several proteins, including those of the plasminogen activator (PA) system. Urokinase-type PA (uPA), which is secreted in inactive form (pro-uPA), plays a key role in ECM degradation. Pro-uPA is converted to its active form after binding to its specific surface receptor, uPAR [1,2]. In women with breast cancer, uPA appears to promote cancer invasion and metastasis [3] through degradation of the ECM, stimulation of angiogenesis, alteration in cell migration and adhesion [4], and inhibition of apoptosis [5].

The total involvement of plasminogen activators in cancer, however, is not that clear. In addition to demonstrated negative effects, PAs apparently play a positive role in certain aspects of the cancer process. For example, PAs induce antiangiogenic activity *in vitro *and in patients with cancer [6]. Several clinical studies have shown associations between high tissue-type PA expression and activity and a favorable prognosis in breast cancer [7]. In a mouse mammary cancer model, induced uPA expression delayed tumor progression and had antiangiogenic and antiproliferative effects. Tumors expressing proteolytically inactive uPA mutants grew faster than tumors overexpressing proteolytically active uPA, suggesting that the inhibitory actions were mediated by uPA's protease activity [8].

Plasminogen activator activity is inhibited by plasminogen activator inhibitor-1 (PAI-1) [9]. While uPA has both positive and negative actions in cancer, PAI-1 promotes breast cancer invasion and metastasis. Deficient PAI-1 expression in mice prevented local invasion and tumor vascularization of transplanted malignant keratinocytes. When PAI-1 expression was restored, invasion and associated angiogenesis were also restored, suggesting that host-produced PAI-1 is essential for cancer cell invasion and angiogenesis [10]. PAI-1 promotes angiogenesis by directly inhibiting proteases [11], suggesting that excessive plasmin proteolysis may prevent the assembly of tumor blood vessels. Possible mechanisms by which PAI-1 promotes breast cancer include prevention of excess ECM degradation, modulation of cell adhesion, a role in angiogenesis, and the stimulation of cell proliferation [3]. It therefore appears that the association of uPA and PAI-1 expression with breast cancer is complex.

Two hypotheses proposed for the effects of PAs on tumor invasiveness [8] are that enhanced proteolytic activity of uPA may result in cleavage of tumor stromal proteins into peptides that inhibit angiogenesis and/or proliferation, or that plasmin generated by uPA activity activates other stromal proteases, leading to tumor matrix disruption.

Celecoxib is an anti-inflammatory agent known to have antiangiogenic [12] and antiproliferative properties [13]. The agent has been shown to prevent [14] and treat mammary tumors in animal models [15]. We observed that celecoxib blocks prostaglandin (PG)E_2 _in high risk women and in women with breast cancer [16]. Celecoxib has been reported to inhibit uPA production in MDA-MB-231 breast cancer cells [17].

We previously reported that PGE_2_, a small lipid associated with breast tumor development, decreased in proportion to circulating celecoxib concentration in postmenpausal women taking the 400 mg twice daily (bid) dose [18]. Our previous experience using celecoxib to inhibit eicosanoid production, the fact that celecoxib affects uPA production and the conflicting findings regarding the role of uPA and PAI-1 in tumor progression caused us to investigate the expression of uPA and PAI-1 in the breast and uPA systemically before and after treatment with low (200 mg bid) and high (400 mg bid) dose celecoxib.

## Methods

### Subject recruitment

Between October 2001 and December 2004, women were provided an Institutional Review Board approved protocol and required to give written informed consent in order to enroll in the study. Subjects enrolled during the first half of the study received 200 mg celecoxib bid, and those enrolled during the second half received 400 mg bid. Subjects evaluated had to be ≥ 18 years old and be at increased breast cancer risk, based on the subject having either a Gail model risk of developing IBC in a 5 year period of > 1.66%, or previously treated DCIS or IBC (now finished with treatment and free of disease).

Pregnant and lactating women were not eligible. Women could not have been currently on nonsteroidal antiinflammatory drugs (NSAIDs), a cyclo-oxygenase-2 (COX-2) inhibitor, warfarin, or have taken such a medication within two weeks of enrollment. Subjects could not have a significant history of peptic ulcer disease, upper gastrointestinal bleeding, asthma, or be allergic to sulfonamides or NSAIDs. A complete blood count, serum electrolytes and liver panel performed within two months of enrollment had to be within normal limits. Subjects were recruited from the breast evaluation clinics at the University of Missouri-Columbia.

### Intervention

Celecoxib pills were taken for 14 days. The treatment period was chosen to be short enough to maximize compliance yet long enough to see a biomarker effect, based on celecoxib's half life of 11 hours. Compliance was assessed through the count of returned pills. All subjects were required to have taken at least 80% of the prescribed medication.

### Specimen collection

NAF was collected using a modified breast pump as previously described [19,20]. Briefly, the breast was warmed with moist heated towels for 5–10 minutes, subsequently massaged from the chest wall toward the nipple while a health care professional provided suction using a modified breast pump. The sample was collected into capillary tubes and stored at -80°C until analysis. NAF volume was measured using a metric ruler. We have determined that one mm in the tube corresponds to approximately one μL of NAF. NAF was collected from only one breast, and NAF from the same breast was analyzed before and after treatment.

Baseline NAF and blood collection were performed prior to the ingestion of celecoxib. Eight mL of blood were also collected from the subject in a tube containing heparin, the blood centrifuged for 10 min at 1600 rpm, and the plasma fraction decanted and stored at -80°C until analysis. All women had NAF and plasma collected within 24 hrs of their last dose of celecoxib, with an average of approximately 12 hrs. The half life of the medication is 11.5 hrs.

### Specimen analysis

#### uPA and PAI-1

uPA and PAI-1 enzyme linked immunosorbant assay kits were obtained from American Diagnostica, Inc. (Stamford, CT). uPA and uPAI-1 levels were determined according to the kit manufacturer's instructions. Briefly, 100 μL of standard and samples were pipetted into the microplate wells that were coated with mouse monoclonal antibodies respectively specific for uPA and PAI-1 and incubated overnight at 4°C. A washing procedure was performed 4 times to remove unbound proteins with washing buffer. Enzyme-linked antibodies specific for each factor were added to the wells and incubated for 1 h at room temperature. The wells were washed again, 100 μL diluted enzyme conjugate (streptavidin conjugated horseradish peroxidase) was pipetted into wells and incubated for 1 h at room temperature, then washed again. Substrate reagent (100 μL) was added to each well and incubated for 1 h at room temperature, followed by a stop solution (0.5 N sulfuric acid). Absorbance values were measured at 450 nm for uPA and uPAI-1 using a microplate reader. The detection limits were 10 pg/mL for uPA and 125 pg/mL for PAI-1. To standardize uPA and PAI-1 expression in NAF, total protein was measured for each sample with a BCA protein assay kit (Pierce Chemicals, Rockford, IL), and results reported as pg of uPA or PAI-1 per mg total NAF protein.

#### PGE_2_

NAF and plasma samples were analyzed by immunoassay for their PGE_2 _content as per the manufacturer's instructions (R&D Systems, Minneapolis, MN). The kit uses a monoclonal antibody to PGE_2 _to competitively bind the PGE_2 _in the standard or sample. Briefly, samples were diluted in 100 μL assay buffer supplied by the manufacturer, pipetted into appropriate wells, incubated for 18–24 hrs at 4°C, washed, substrate solution added, followed by 1 hr incubation, and absorbance measured at 405 nm.

A standard curve was prepared using serial dilutions of PGE_2_. A linear regression equation was created from standards of known PGE_2 _concentration, and PGE_2 _concentrations of unknown samples fit to the standard curve regression equation, corrected for aliquot volume and expressed as nanograms of PGE_2_/mL of original sample. Whenever possible, NAF and plasma samples were run in duplicate and the average of the two values was reported. The goodness of fit of the standard curve, R^2^, for NAF samples was 0.999. The goodness of fit was similar for the plasma samples.

#### Celecoxib

Celecoxib was analyzed in plasma using a modification of the technique of Schonberger *et al*. [21] by combining 250 μL aliquots of plasma with an equal volume of distilled water and adding 500 μL ethanol to precipitate the protein. Spiked plasma samples were prepared by combining 250 μL blank plasma with 250 μl distilled water, 20 μL of 10 ppm celecoxib in ethanol and 480 μL of ethanol. Samples were vortexed and then centrifuged at 13,000 rpm for 5 min. A 500 μL aliquot of the supernatant was combined with 1.5 mL distilled water and applied to a Waters 3 mL C_18 _Sep-Pac-Vac disposable cleanup column (Waters, Milford, MA) which was preconditioned with 2 mL methanol and then 2 mL distilled water. Cleanup columns were washed with 2 mL distilled water and vacuum dried for 15 minutes. Celecoxib was eluted with 4 mL methanol and the sample eluants taken to dryness. Samples were reconstituted in 1.0 mL methanol:water (80:20) for HPLC analysis.

HPLC analysis was performed on an Hitachi HPLC system (Hitachi Instruments, Inc., San Jose, CA) which consisted of an L7100 pump, with an L7200 autosampler (20 ul injected), and fluorescence detection with an L7480 fluorescence detector (excitation 240 nm, emission 380 nm). The system was controlled, data acquired and processed using an Hitachi D-7000 data acquisition package with Concert Chrom software on a microcomputer. A Phenomenex Hypersil BDS C_18 _analytical column (250 × 4.6 mm, 5 um) (Phenomenex, Torrence, CA) and a Phenomenex Securityguard C_18 _precolumn with a mobile phase of acetonitrile:water (70:30) was used at a flow rate of 1 mL/min. Celecoxib was kindly provided by Pfizer Corporation, New York, NY. A primary standard of celecoxib (2,000 ppm) was prepared in acetonitrile. Working standards (100, 200, 500 ppb) were prepared in methanol:water (80:20). Plasma samples spiked with celecoxib had recoveries greater than 95%.

### Toxicity

Among women taking celecoxib 200 mg bid, two subjects experienced side effects (one nausea, the second a cough), with both resolving spontaneously. There were no dropouts in the 200 mg bid group. Among women enrolled in the 400 mg bid group, 11 experienced side effects from celecoxib, four of whom dropped out. Of the four who dropped out, the side effects (edema in two, diarrhea in one, and heart palpitations in one) resolved shortly after stopping celecoxib. Among the remaining seven subjects, the side effects: diarrhea, nausea, rash, altered taste, urinary urgency, sweating, and muscle tension, all resolved spontaneously.

### Statistical analysis

Median values of continuous variables were computed for the various groups of subjects. Due to the potential non-normality of the data, ranking procedures were used for all analyses with continuous variables. The Wilcoxon Rank Sum Test was used to compare independent groups. Examples of these comparisons include comparing pre- and postmenopausal women. The Wilcoxon Signed Rank Test was used to make within group comparisons such as comparing pre treatment to post treatment.

We are interested in the change which occurs from pre to post treatment. Because of the potential skewness of the data, we report medians rather than means. The delta (Δ) reported in Table 2 is the median of the post – pre treatment differences. It should be noted that the median of these differences is seldom equal to the difference (post – pre) in the medians. Thus, the Δ reported in Table 2 could be negative and the difference in the medians positive.

**Table 2:** Median concentrations in NAF of uPA and PAI-1 (pg/mg), PGE_2 _(ng/mL) and in plasma of celecoxib (ng/mL) based on celecoxib dose and menopausal status^1^

**Dose**	**N**	**Baseline**	**After Tx**	**Δ**	**P value**	**Celecoxib**
**200 mg bid^2^**						
**uPA**						
Overall	9	422.0	375.2	-19.6	0.10	346.0
Premen	2	133.6	130.1	-3.5	0.50	231.8
Postmen	7	645.1	402.4	-57.0	0.22	728.2
**PAI-1**						
Overall	8	709.0	194.4	-26.7	0.56	288.1
Premen	2	2672	1821	-850.8	0.99	231.8
Postmen	6	709.0	194.4	-26.7	0.99	479.1
**PGE_2_**						
Overall	9	8.71	13.34	-1.04	0.91	346.0
Premen	2	25.45	25.96	0.52	0.99	231.8
Postmen	7	8.71	9.80	-1.04	0.81	728.2
**400 mg bid^2^**						
**uPA**						
Overall	11	372.6	321.6	22.0	0.46	184.0
Premen	5	372.6	321.6	11.0	0.99	0
Postmen	6	409.1	382.4	28.2	0.31	1141.8
**PAI-1**						
Overall	5	2401	827.8	0	0.62	156.8
Premen	1	281.6	344.7	63.1	-	0
Postmen	4	2416	1437	-801.7	0.50	789.9
**PGE_2_**						
Overall	11	12.32	13.45	-1.77	0.96	184.2
Premen	5	12.32	51.24	21.95	0.31	0
Postmen	6	14.79	13.22	-6.41	0.16	1141.8

1: N: sample size; NAF: nipple aspirate fluid. NS: not significant. Units reflect pg uPA or PAI-1/mg total protein. Δ reflects the median of the change measured in each subject (after treatment value – before treatment value) due to treatment. Celecoxib level is from plasma collected after treatment.

2: Not all NAF samples were sufficient to analyze all proteins; bid: twice daily; premen: premenopausal; postmen: postmenopausal

## Results

### Subjects

Of 42 women evaluated, 17 took celecoxib 200 mg bid and 25 celecoxib 400 mg bid . Matched (pre and post treatment) plasma was available from all subjects. Matched NAF was available for analysis from approximately half of the subjects. All subjects reported that they took over 80% of the pills they were administered (Table 1).

**Table 1:** Demographics

	**Celecoxib Dose**	
	200 mg twice daily NAF (plasma)	400 mg twice daily NAF (plasma)
Samples	9 (17)	11 (25)
Age (years)		
Median	46 (51)	51 (50)
Range	23–67 (23–68)	30–68 (30–81)
Menopause		
Pre	2 (4)	5 (11)
Post	7 (13)	6 (14)

### Celecoxib concentrations in ng/mL

At the end of treatment, celecoxib was detectable in the plasma of 14 of 17 participants (82%) in the 200 mg group, and 19 of 25 participants (76%) in the 400 mg group. The limit of detection of the assay was 100 ng/mL. Of samples in which celecoxib was detectable, values ranged from 117.6 to 2281.2 ng/mL in the 200 mg group and from 156.8 to 16403.1 ng/mL in the 400 mg group. Levels trended (p = 0.12) higher in women taking 400 mg (median 1025.0 ng/mL) compared to 200 mg bid (median 227.3 ng/mL), and in post- (534.6 ng/mL) compared to premenopausal (227.3 ng/mL) women (p = 0.13).

### uPA levels in postmenopausal women increase, while PAI-1 and PGE2 decrease, after treatment with high dose celecoxib

We first evaluated the change in uPA, PAI-1 and PGE_2 _concentration in the NAF of each participant after low and high dose celecoxib administration (Table 2). The delta (Δ) in Table 2 represents the median change due to celecoxib intervention. uPA trended downward with low dose and upward with high dose treatment. PAI-1 trended downward at both celecoxib doses. PGE_2 _concentrations also tended to decrease overall at both doses of celecoxib but the values measured in premenopausal women were slightly increased. This coincided with relatively low plasma concentrations of celecoxib. The changes in uPA, PAI-1 and PGE_2 _were not significant at either dose for pre- or postmenopausal women.

We next evaluated the change in plasma uPA and PGE_2 _concentration after low and high dose celecoxib administration (Table 3). PAI-1 was not evaluated in plasma due to inadequate samples available. Similar to NAF, uPA levels in plasma trended downward with low dose and upward with high dose treatment. Plasma PGE_2 _changed little after treatment, with a small move upward with low dose and downward move with high dose therapy. The change in neither uPA nor PGE_2 _was significant for either pre- or postmenopausal women.

**Table 3:** Median concentrations in plasma of uPA (pg/mL), PGE_2 _(ng/mL) and celecoxib (ng/mL) based on celecoxib dose and menopausal status^1^

**Dose**	**N**	**Baseline**	**After Tx**	**Δ**	**P Value**	**Celecoxib**
**200 mg bid^2^**						
**uPA**						
Overall	17	941.4	955.0	-20.7	0.85	233.7
Premen	4	886.9	1031.8	36.0	0.62	195.3
Postmen	13	994.0	945.6	-20.7	0.64	233.7
**PGE_2_**						
Overall	17	0.209	0.206	0.005	0.96	233.7
Premen	4	0.244	0.221	0.100	0.99	0
Postmen	13	0.204	0.195	0.005	0.84	233.7
**400 mg bid^2^**						
**uPA**						
Overall	25	908.3	910.7	17.5	0.8	696.0
Premen	11	864.9	821.0	17.5	0.8	227.3
Postmen	14	1036.1	992.4	7.9	0.7	1024.9
**PGE_2_**						
Overall	25	0.280	0.203	-0.009	0.93	696.0
Premen	11	0.375	0.366	-0.001	0.77	227.2
Postmen	14	0.280	0.175	-0.023	0.90	1025.0

1: N: sample size; NAF: nipple aspirate fluid. Units reflect pg uPA/mL plasma volume. Δ uPA reflects the median of the change measured in each subject (after treatment value – before treatment value) due to treatment. Celecoxib level is from plasma collected after treatment.

2: bid: twice daily; premen: premenopausal; postmen: postmenopausal.

### Effect of circulating celecoxib concentrations on biomarker change

**Figure 1:**
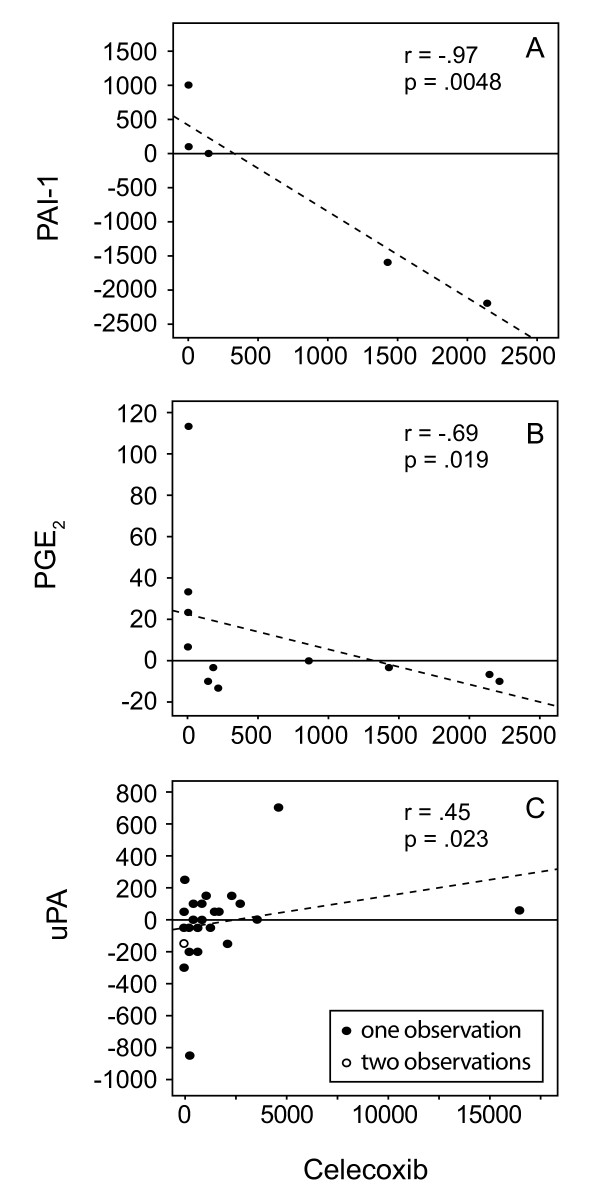
**Celecoxib concentration predicts on biomarker change**. In women taking celecoxib 400 mg bid, the change in PAI-1 (pg/mg) (A) and PGE_2 _(ng/mL) (B) in NAF and uPA (pg/mL) (C) in plasma correlated with celecoxib concentration (ng/mL).

Considering subjects in both dosage groups, there was an inverse correlation between celecoxib concentration and change in NAF PGE_2 _(r = -.42, p = .06, n = 20) and plasma uPA (r = .38, p = .012, n = 42) post treatment, which was not observed for the other markers. There were no significant associations between uPA, PAI-1 or PGE_2 _in NAF or plasma and celecoxib concentration in women receiving low dose celecoxib. In women taking the higher dose, both PAI-1 (r = -.97, p = .0048, n = 5) and PGE_2 _(r = -.69, p = .019, n = 11) in NAF and uPA in plasma (r = .45, p = .023, n = 25) were correlated with celecoxib concentration (Figure 1).

## Discussion

We carried out experiments to investigate whether the COX-2 inhibitor celecoxib at a dose of either 200 mg bid or 400 mg bid could significantly affect endogenous uPA, PAI-1 or PGE_2 _production in women at increased risk for breast cancer. The same NAF and plasma samples were used to measure all three markers so that we could determine celecoxib's coordinate effect on them. This study builds on our initial study, which found that celecoxib 400 mg bid decreased PGE_2 _levels in NAF and/or plasma in women with newly diagnosed breast cancer and in high risk women [16].

We previously observed that uPA and PAI-1 are concentrated in NAF compared to plasma [22]. This fact, as well as the fact that NAF is breast specific, undiluted by the contribution from other organs, suggests that it may be a better physiologic fluid than plasma to identify breast cancer biomarkers.

uPA expression trended downward among women receiving the lower celecoxib dose and upward among those receiving the higher dose, suggesting a dose-dependent effect of the medication. Sample size may have limited our ability to detect a significant effect. The different uPA response was more noted in post- than in premenopausal women, in whom celecoxib concentrations tended to be higher. Similarly, we previously observed that high (but not low) dose celecoxib significantly decreased PGE_2 _levels in post- but not premenopausal women, in whom higher circulating celecoxib levels were noted [16]. In the current study a similar trend was noted for PGE_2_. Sample size may have limited our ability to detect a significant effect.

Mice prone to the development of intestinal adenomas that were made deficient in uPA were found to upregulate COX-2 expression [23]. It has been shown that PAs generate endogenous angiogenesis inhibitors, and mice with tumors that overexpressed uPA demonstrated tumor growth inhibition, fewer lung metastases and prolonged survival compared to controls [8]. There is evidence that PAI-1, an inhibitor of uPA, may promote angiogenesis [10]. It was found that celecoxib was more effective than the combination of celecoxib plus the PA inhibitor amiloride in reducing the number of pulmonary metastases, with metastases suppressed by 67% with amiloride alone, 98% with celecoxib alone and 81% with the combined regimen. In our own subjects, medication dose provided hints as to the effect of celecoxib, but the effect was masked by individual differences in celecoxib metabolism. On the other hand, evaluating the circulating level of celecoxib demonstrated the effect of the agent, and consistent with preclinical reports discussed above, NAF levels of PAI-1 and PGE_2 _decreased in relation to increasing celecoxib concentrations, while uPA plasma levels increased in relation to increased circulating celecoxib (Figure 1).

## Conclusion

Patients receiving celecoxib 200 mg bid demonstrated decreased uPA concentrations in NAF and plasma whereas patients receiving celecoxib 400 mg bid demonstrated increased concentrations of uPA in both NAF and plasma. PAI-1 was more decreased in the NAF of patients receiving the 200 compared to the 400 mg bid celecoxib dose. PGE_2 _concentrations trended lower in both NAF and plasma after celecoxib treatment. PAI-1 and PGE_2 _decreased in NAF and uPA increased in plasma in relation to the circulating level of celecoxib, which may better reflect the action of the agent than dosage, since individuals metabolize celecoxib at different rates. These findings are consistent with animal studies which suggest that, unlike in subjects with invasive breast cancer (in whom high levels of uPA are associated with a poor prognosis [24]), in high risk women the upregulation of uPA (in association with downregulation of PAI-1 and PGE_2_) may have a breast cancer chemopreventive effect.

## Abbreviations

bid: twice daily; COX: cyclooxygenase; ECM: extracellular matrix; HPLC: high performance liquid chromatography; NAF: nipple aspirate fluid; NSAIDs: nonsteroidal anti-inflammatory drugs; PG: prostaglandin; PAI: prostaglandin inhibitor; uPA: urokinase-type plasminogen activator.

## Competing interests

The authors declare that they have no competing interests.

## Authors' contributions

ERS designed the study, enrolled subjects, and performed the majority of manuscript preparation. WQ and WZ conducted uPA, PAI-1 and PGE_2 _analyses. JEH performed the statistical analyses, GR and YCC conducted the celecoxib analyses. FM and JTF assisted with manuscript preparation and critique. BK assisted with manuscript preparation and data gathering. All authors read and approved the final manuscript.

## Pre-publication history

The pre-publication history for this paper can be accessed here:


